# Novel optical photothermal infrared (O-PTIR) spectroscopy for the noninvasive characterization of heritage glass-metal objects

**DOI:** 10.1126/sciadv.abl6769

**Published:** 2022-03-04

**Authors:** Andrea Marchetti, Victoria Beltran, Gert Nuyts, Ferenc Borondics, Steven De Meyer, Marina Van Bos, Jakub Jaroszewicz, Elke Otten, Marjolijn Debulpaep, Karolien De Wael

**Affiliations:** 1 AXES Research Group, University of Antwerp, Groenenborgerlaan 171, 2020 Antwerp, Belgium.; 2 NanoLab Centre of Excellence, University of Antwerp, Groenenborgerlaan 171, 2020 Antwerp, Belgium.; 3 Synchrotron Soleil, L’Orme des Merisiers, Saint-Aubin, BP48, 91192 Gif-sur-Yvette CEDEX, France.; 4 Royal Institute for Cultural Heritage (KIK-IRPA), Parc du Cinquantenaire 1, B-1000 Brussels, Belgium.; 5 Warsaw University of Technology, Faculty of Materials Science and Engineering, ul. Wołoska 141, 02-507 Warsaw, Poland.

## Abstract

Optical photothermal infrared (O-PTIR) is a recently developed molecular spectroscopy technique that allows to noninvasively obtain chemical information on organic and inorganic samples at a submicrometric scale. The high spatial resolution (≈450 nm), lack of sample preparation, and comparability of the spectral results to traditional Fourier transform infrared spectroscopy make it a promising candidate for the analysis of cultural heritage. In this work, the potential of O-PTIR for the noninvasive characterization of small heritage objects (few cubic centimeters) is demonstrated on a series of degraded 16th century brass and glass decorative elements. These small and challenging samples, typically encountering limitations with existing noninvasive methods such as macroscopic x-ray powder diffraction and μRaman, were successfully characterized by O-PTIR, ultimately identifying the markers of glass-induced metal corrosion processes. The results clearly demonstrate how O-PTIR can be easily implemented in a noninvasive multianalytical strategy for the study of heritage materials, making it a fundamental tool for cultural heritage analyses.

## Teaser

Optical photothermal infrared spectroscopy allows noninvasive high-resolution chemical characterization of heritage materials.

## INTRODUCTION

The most crucial information needed to unravel history, conservation state, and potential risks for cultural heritage is the nature and distribution of different elements and molecules in the objects. However, extracting this information is generally extremely complex because of the heterogeneity of the materials, often down to a submicrometric scale.

Several analytical techniques have been developed and applied over the years that can answer these questions. Still, given the relevance and uniqueness of the objects to be preserved, the analytical methods that do not prejudice the integrity of the object nor damage it irreversibly (noninvasive and nondestructive) are generally preferred. While different noninvasive options are available for the high-resolution elemental characterization of heritage objects (*1*, *2*), the existing molecular speciation and imaging techniques still have strong limitations, particularly when it comes to noninvasive and nondestructive characterization of materials with high spatial resolution (Table 1).

**Table 1.** Relevant features of the high spatial resolution molecular speciation techniques most commonly used for the analysis of cultural heritage objects (SR-μXRPD, SR-μFTIR, μRaman, and AFM-IR) and of the novel O-PTIR spectroscopy.

	**SR-μXRPD (*1*, *53*–*55*)**	**SR-μFTIR (*56*–*58*)**	**μRaman (*59*–*61*)**	**AFM-IR (PTIR) (*62*, *63*)**	**O-PTIR**
Invasive	No*	No^†^	No^‡^	Yes	No^‡^
Sample preparation	Complex^§^	Complex§	Not needed	Complex^§^	Not needed
Maximum spatial resolution	≈50 × 50 nm^2^	≈10 × 10 μm^2^^||^	≈1 × 1 μm^2^^¶^	≈20 × 20 nm^2^	**≈**450 × 450 nm^2^
Materials detected	Crystalline only	Crystalline and amorphous (IR active)	Crystalline and amorphous (Raman active)	Crystalline and amorphous (IR active)	Crystalline and amorphous (IR active)
Risk of beam damage (destructive)	High	Low	Medium	Low	Low
Fluorescence interference	No	No	Yes	No	No
Benchtop equipment	No	No	Yes	Yes	Yes

*Noninvasive applications limited to flat, convex, and/or very thin objects (SR-μXRPD).

†Noninvasive applications limited to very flat objects.

‡Sampling needed for large objects (larger than few square centimeters).

§Nonflat objects need to be prepared in cross section or, more often, in thin sections.

‖Diffraction limited (i.e., dependent on the wavelength of the incident light).

¶Depending on the wavelength of the visible laser and on the focusing optics.

To overcome the limitations of the traditional methods listed in Table 1, in this work, we test a novel analytical technique and apply it to the noninvasive characterization of small (few cubic centimeters) heritage objects. The technique in question is the recently developed optical photothermal infrared (O-PTIR) spectroscopy.

O-PTIR exploits the photothermal effect induced in infrared (IR)–active samples by the irradiation with a tunable IR laser, which is then measured using a visible laser. The collected data are similar to transmission Fourier transform infrared (FTIR) spectra, allowing for direct comparison with the literature (*3*–*5*). One of the main advantages of the technique is not needing any complex sample preparation, unlike traditional FTIR. Small objects can, in fact, be analyzed in a completely noninvasive and nondestructive manner. Moreover, the use of a visible laser probe increases the achievable spatial resolution (≈450 nm) by overcoming the diffraction limits intrinsic to traditional FTIR spectroscopy. O-PTIR was recently applied on cultural heritage materials by some of the authors (*6*).

However, while notable results were obtained in terms of submicrometer molecular characterization of heritage materials, the analyses were performed on samples extracted from a historical painting and prepared in thin sections. The potentially noninvasive and direct (no sample preparation) nature of the technique is, therefore, yet to be demonstrated.

In this work, the possibility of performing direct O-PTIR analysis of small cultural heritage objects without sampling or preparation, while still obtaining high spatial resolution chemical information, is demonstrated for a series of corroded brass objects and weathered glass beads from the 16th century Enclosed Gardens in the collection of the Museum Hof Van Busleyden (Mechelen, BE) (*7*, *8*). These reliquary altarpieces were kept for centuries in the convent of the Augustinian nuns inside the city walls of Mechelen, protected from the outdoor environment in the cells of the sisters but likely exposed to high humidity levels because of the lack of heating. While most materials in the Enclosed Gardens underwent severe transformations over time (e.g., faded and embrittled textiles, weathered glass beads, corroded silver objects), the brass elements in these reliquaries mostly survived in a pristine state. The small selection of heavily corroded brass elements considered in the present manuscript therefore represents an exception to the general conservation state of the hundreds of brass objects in the Enclosed Gardens. The factors that induced the corrosion are still unknown; thus, identifying the reason for the exceptionally poor conservation state of these metal objects and of the glass beads considered is fundamental to correctly preserve the altarpieces and prevent potential future hazards. A thorough characterization of the original materials and their corrosion products is therefore crucial. However, the complex three-dimensional (3D) shape of the objects under examination and the small amounts and high heterogeneity of the degradation products pose a complex analytical challenge, creating the need for a versatile technique such as O-PTIR.

In the first place, areas of interest on the surface of the samples were selected on the basis of exploratory macro x-ray fluorescence (MA-XRF), micro XRF (μXRF), and micro–computed tomography (μCT) analyses. Then, a high-resolution characterization of the corrosion products was performed by means of O-PTIR and complementary noninvasive [scanning electron microscopy with energy dispersive x-ray spectroscopy (SEM-EDX), μRaman, and macroscopic x-ray powder diffraction (MA-XRPD)] and invasive techniques [synchrotron radiation micro FTIR (SR-μFTIR)]. The versatility and submicrometer resolution of O-PTIR, lastly, allowed fundamental information to be unraveled that would have remained hidden with MA-XRPD and μRaman analyses, ultimately demonstrating that a glass-induced corrosion process is responsible for the poor conservation state of the brass elements considered. These notable results confirm the potential for O-PTIR to become a fundamental tool in the future of cultural heritage analyses.

## RESULTS

### Samples

To shed light on the severe degradation affecting the selection of brass elements considered, one heavily corroded sequin (leaf-shaped hanging decorative element) (1S, Table 2 and table S1) and three corroded wires were analyzed (1W to 3W, Table 2 and table S1). In addition, two dark blue glass beads, with clearly degraded areas showing a partially detached brown-iridescent layer, were also studied (1B and 2B, Table 2 and table S1). All the samples analyzed, both glass and metal, showed different amounts of visually similar white and green-blue degradation products on their surface (fig. S1). Glass and brass might have originally been in close contact, but, given the extreme corrosion and loss of structural properties of the metallic elements, the original position is not always certain.

**Table 2.**
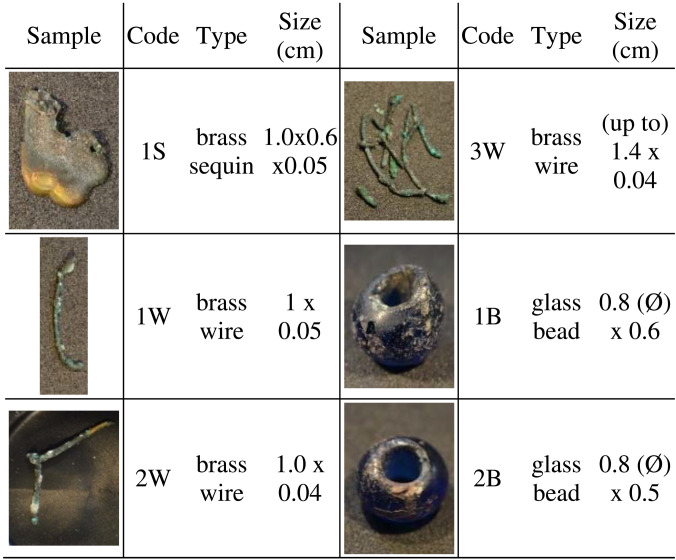
Overview of the historical objects considered in this study. Metal sequin (S), metal wires (W), and glass beads (B).

### Exploratory elemental and CT analyses

MA-XRF, μXRF, and μCT were first used to identify the areas of interest on the sequin, wires, and beads (see more details in the Supplementary Materials). These exploratory analyses allowed the following: (i) highlight the presence of elements, in the corrosion products on the surface of the metal objects (sequin and wires), traceable to the composition of the glass (i.e., K), and (ii) identify elements, present in the corrosion products on the surface of the glass beads, likely ascribable to the brass composition (i.e., Cu and Zn).

These results support the hypothesis of a direct chemical interaction between the glass and the metal objects. In particular, the systematic presence of K, often mixed with Zn (figs. S2 and S3), on the surface of the corroded brass elements, is likely a result of a glass-induced corrosion process of the metal (*9*). Alkaline cations such as K^+^ (and Na^+^) can, in fact, leach out from the silica network during the weathering of glass through a cation exchange process initiated by the condensation of atmospheric moisture (*10*–*13*). This process creates a transformed surface gel layer with a lower density than the bulk glass and causes a local increase in the pH in the solution deposited on the glass surface (*14*, *15*). If this alkaline liquid film comes into contact with a metal (e.g., when glass and metal objects are in close proximity), this can promote corrosion and the formation of degradation products, which are often mixed metal salts containing both alkali cations from the glass and additional species from the corroding metal (*9*, *16*–*19*). Zn and K mixed salts (possibly carboxylates) particularly have been recently described by Fischer *et al.* (*17*) as products of a glass-induced metal corrosion on the surface of historical brass objects.

The hypothesis of K^+^ originating from the glass beads is clearly supported by the chemical composition of the glass, because both glass beads are made of a potash-rich glass [fig. S4; see more details in the Supplementary Materials section “Supplementary experimental results (exploratory elemental and CT analyses)”] and by the poor conservation state of the beads. A partially detached iridescent transformed layer visible on the surface (fig. S5), in fact, suggests that cation leaching processes took place (*13*, *15*).

In a similar fashion, the enrichment of Cu and Zn in the corrosion products on the surface of the weathered glass beads (fig. S4) and particularly in areas where the contact with the metal would have been more probable (on the central shaft and inner rim; fig. S6) also supports the hypothesis of an interaction between brass and glass.

Nonetheless, it needs to be mentioned that the presence of K^+^ in the corrosion products of a copper alloy is not sufficient, on its own, to confirm a chemical interaction with glass. This cation, in fact, often occurs in airborne particulate matter (*20*, *21*) and might therefore be present on the metal because of deposition and soiling (*22*). Similarly, Cu and Zn might be also present in the bulk composition of the glass, particularly linked to coloring agents (*23*, *24*), and therefore might not be associated to a chemical interaction with brass. Hence, to unequivocally confirm the hypothesis of a glass-induced metal corrosion in the Enclosed Gardens by noninvasive techniques, further chemical evidence is needed. To obtain this information, two traditional methods such as XRPD and Raman spectroscopy were first tested in a noninvasive configuration (MA-XRPD and μRaman), followed by the novel O-PTIR spectroscopy.

### Shortcomings of traditional analytical methods for the noninvasive analysis of the objects

The analyses by means of XRPD and μRaman spectroscopy, techniques commonly used for the characterization of glass-metal corrosion products (*9*, *16*, *17*, *25*, *26*), did not lead to clear and conclusive results (fig. S7). This is a direct consequence of the specific drawbacks and limitations intrinsic to the techniques used. Complex 3D geometries present, in fact, a serious challenge to analyze with the noninvasive MA-XRPD scanner used in this study (*27*). Furthermore, the relatively large spot size of the technique in this configuration (1 × 0.150 mm) and high detection limits (few weight % depending on the material) hindered the identification of the small and localized amounts of corrosion products observed on the glass and wire samples (fig. S7). On the other hand, μRaman spectroscopy showed limitation mainly related to the extreme sensitivity of the corrosion products to the laser source. The use of a laser power higher than 1%, in fact, caused clear damages to the objects (fig. S8), while lower laser powers resulted in low signal-to-noise ratios, hindering a detailed interpretation of the experimental results (fig. S7).

### O-PTIR for the identification of glass-induced corrosion products supported by complementary analyses

The possibility to noninvasively and nondestructively characterize the surface of both metal and glass samples makes O-PTIR highly attractive for the given set of objects. Nonetheless, because O-PTIR uses a visible laser in a similar fashion to μRaman, this could result to radiation damage on sensitive materials such as the ones under examination. This possibility was investigated first by performing a series of O-PTIR replicate measurements on areas of a wire sample (sample W3) characterized by a high sensitivity toward visible light. A thorough observation with optical microscopy (OM) before and after the analysis clearly confirmed the lack of visible damage for the materials in exam, as opposed to μRaman spectroscopy (fig. S8). This is likely a direct result of the low power of the visible laser used by O-PTIR compared with μRaman.

**Fig. 1.**
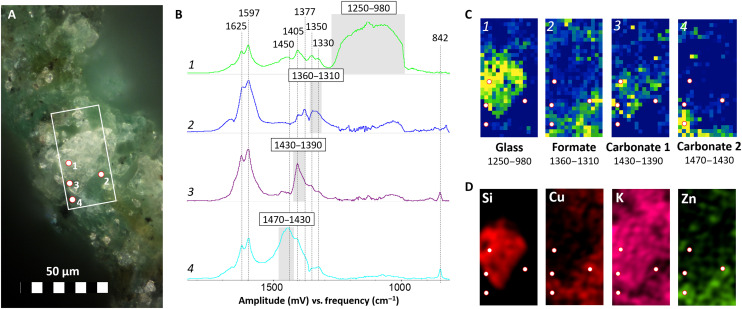
High-resolution O-PTIR molecular imaging of degradation products on the surface of the degraded brass sequin (1S). (**A**) OM photomicrograph of the area in analysis, in evidence the location of the representative spectra shown in (B) (numbered points) and of the imaged area (white rectangle). (**B**) Representative spectra of the imaged species and integration range (in gray). (**C**) Corresponding integration maps (30 × 62 μm with a pixel size of 2 μm) with tentative interpretation (integration range in cm^−1^). (**D**) Complementary SEM-EDX elemental imaging of the scanned area.

**Fig. 2.**
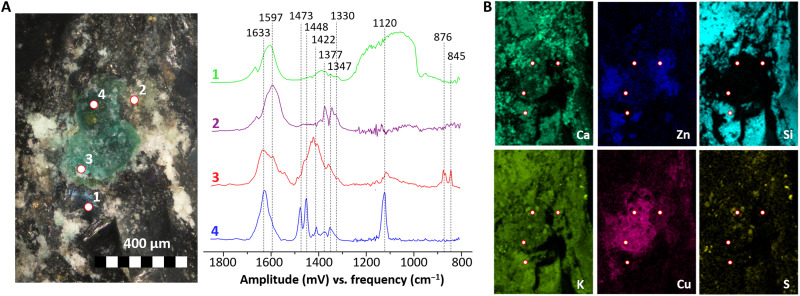
O-PTIR point analysis of degradation products on the surface of a glass bead (sample 1B). (**A**) Locations analyzed (OM photomicrograph) and corresponding spectra. (**B**) Complementary SEM-EDX elemental imaging of the region of interest.

**Fig. 3.**
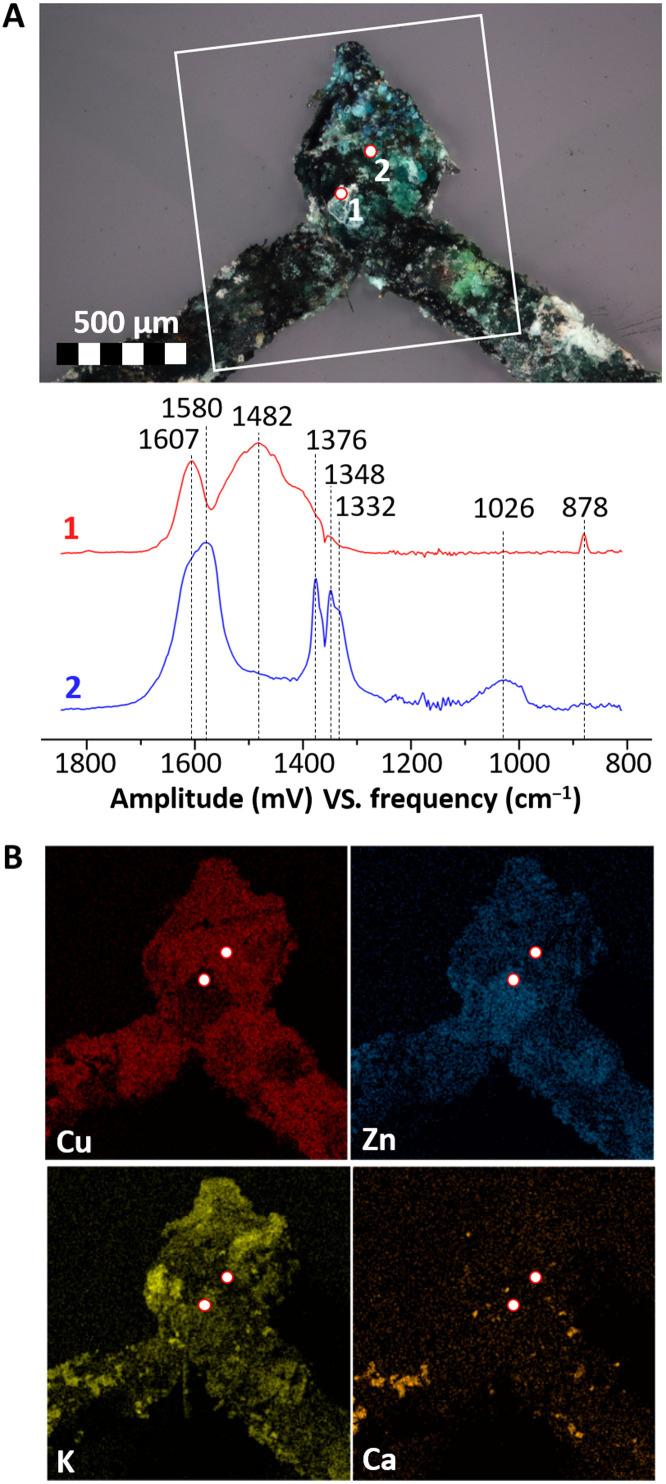
O-PTIR point analysis of degradation products on the surface of a corroded brass wire (sample 2W). (**A**) Locations analyzed (OM photomicrograph) and corresponding spectra. (**B**) Complementary SEM-EDX elemental imaging of the region of interest [white rectangle in (A)].

In Figs. 1 to 3, an overview of the results of O-PTIR and complementary analyses on three different samples (representative of the three categories of objects considered: metal sequin, metal wires, and glass beads) are presented. In this case, a spectral range from 1900 to 800 cm^−1^ was considered, but this can be easily expanded by using a different set of quantum cascade lasers (QCLs). In particular, Fig. 1 shows the analysis of a small area (30 × 62 μm) on the surface of the corroded sequin (1S, fig. S9A), while Figs. 2 and 3 contain the results of point analyses on the surface of a glass bead (1B, fig. S9B) and a corroded wire (2W, fig. S9C) respectively.

The first fundamental result of the O-PTIR analysis is the possibility of recording clear IR spectra on the surface of both metal and glass samples, with high signal-to-noise ratios, a straight baseline, and no spectral artifacts, despite the lack of sample preparation. Moreover, relevant molecular information supporting the hypothesis of a glass-induced corrosion of the brass elements under examination was successfully obtained.

The molecular signature of glass is clearly identified in a particle on the surface of the metal sequin (Fig. 1B, spectrum 1, and Fig. 1C) because of the very broad and intense (slightly saturated) band between 1250 and 980 cm^−1^ [Si-O-Si and Si-O regions; (*28*)]. A very similar spectrum is observed in a visually nondegraded area on the surface of a potash glass bead (Fig. 2A, spectrum 1), which supports the hypothesis of a direct contact between the brass object and this type of glass. The chemical information obtained with O-PTIR goes beyond but clearly agrees with the complementary SEM-EDX elemental analysis (Figs. 1D and 2B), showing higher Si and K contents in the areas identified as glass. The additional bands in the 1650- to 1300-cm^−1^ region hint to the presence of other species (e.g., spectra 2, 3, and 4 in Fig. 1B) overlapping and in close contact with the glass particle. It is noticeable that similar spectral features can be observed in this region on the surface of the glass beads (Fig. 2A, spectrum 1), indicating that the related compounds might be associated to the degradation of glass. In particular, the spectrum agrees with the presence of carboxylate (formate and/or acetate) species (bands at ≈1600 to 1500 and ≈1500 to 1400 cm^−1^ related to C-O asymmetric and symmetric stretching, respectively) and with the presence of carbonates (bands at ≈1450 to 1350 cm^−1^ related to C-O stretching) (*29*–*32*). These compounds have been previously linked to the degradation of glass (*9*, *33*, *34*). In addition, the bands at ≈1625 and ≈1320 cm^−1^ may be linked to the presence of oxalates, which are usually found linked to different types of degradation processes on cultural heritage materials (*35*). The spectral features of oxalates appear in all spectra in Fig. 1, suggesting a presence of these corrosion products in all points analyzed. The results demonstrate that the mentioned compounds may coexist in the same region.

**Fig. 4.**
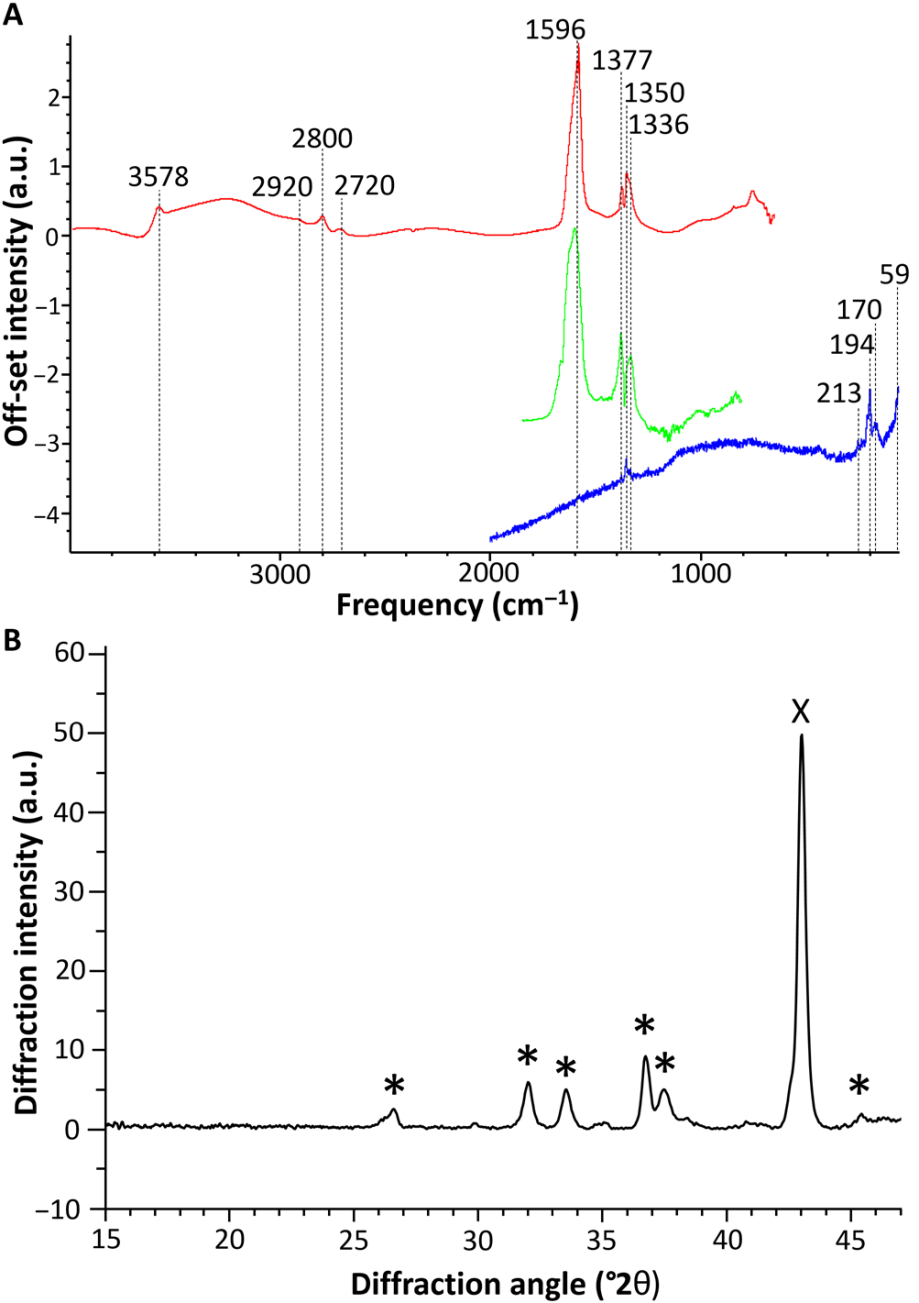
Experimental spectra of basic Cu formate. (**A**) O-PTIR (green), SR-μFTIR (red), and μRaman (blue) on a corroded wire (sample 3W). a.u., arbitrary units. (**B**) XRPD collected on the corroded sequin (sample 1S), average diffractogram for a 3-mm-long line in the degraded area of the sample. In evidence the diffraction peaks associated to Cu_2_(OH)_3_(HCOO) (*) (*9*) and high-Cu brass (X) (*52*).

Spectrum 2 in Fig. 1B is one of the spectra most commonly encountered in the green-bluish areas of the sequin, glass beads, and wires alike (Fig. 2A, spectrum 2; Fig. 3A, spectrum 2; and fig. S10, spectrum 1). Complementary data for this compound were obtained from the SR-μFTIR and μRaman analysis of the corrosion products of one wire (sample W3; Fig. 4A). This compound is more abundant in areas containing Cu and O and close to no Zn and K (Fig. 1, C and D, and fig. S10). The intense peaks in the 1650- to 1580-cm^−1^ and 1400- to 1320-cm^−1^ regions indicate the presence of a carboxylate and, particularly, of a Cu formate (*36*). The strong peak at ≈1600 cm^−1^ is assigned to the asymmetric stretching vibration of the C-O from the carboxylate group, while the peak at 1350 cm^−1^ is assigned to its symmetric stretching vibration (*30*). The band at 1377 cm^−1^ is linked to the in-plane bending of the CH group in the formate moiety, and the band at 2800 cm^−1^, which can be seen in the SR-μFTIR spectrum, to the C-H stretching of the same functional group (Fig. 4A) (*37*). The good match of the Raman peaks at 213, 170, and 59 cm^−1^ with the in-plane lattice vibrations of copper formate tetrahydrate (*38*) confirms this band assignment. In addition, a sharp peak at 3578 cm^−1^ in the SR-μFTIR spectrum indicates that free (non–hydrogen-bonded) OH groups are also present in the corrosion product in analysis (*36*), suggesting the presence of Cu-OH moieties in the lattice. The 194-cm^−1^ vibration in the Raman spectrum can therefore be assigned to the O-Cu-OH out-of-plane bending (together with an overlapped band at 170 cm^−1^) (*39*). The presence of a basic copper formate, and particularly of Cu_2_(OH)_3_(HCOO) (*9*), in the corrosion products was confirmed by the XRPD analysis of the sequin (sample 1S), the only sample flat and large enough to be successfully analyzed with this technique (Fig. 4B).

Basic copper formates are extremely rare as corrosion products on historical metal objects, unless the degradation is catalyzed by the contact with weathered glass (*9*, *17*). The alkaline liquid film formed during the degradation of glass, in fact, favors both the solubilization of the metal alloy and the conversion of formaldehyde [off-gassed during the aging of wood and other organic materials; (*40*)] to formic acid through the Cannizzaro reaction (*17*), ultimately leading to the precipitation of the basic form of the metal formate. In the context of this study, the diffuse presence of basic copper formate on all the samples in analysis (the spectral markers of this compound are visible in all spectra from Figs. 1 to 3) decisively confirms the existence of a chemical interaction between weathered glass and brass. The strong connection between this formate corrosion product and glass is further supported by the close contact observed between these two materials in Fig. 1C, visualized thanks to the high spatial resolution allowed by the O-PTIR analysis.

Spectrum 3 in Fig. 1B is tentatively assigned to a carbonate species; in particular, the band at ≈1400 cm^−1^ is likely related to the stretching and the one at ≈842 cm^−1^ to the bending of the CO_3_^2−^ group (*41*). Given the overlap of the bands with the signal from other species, the identification of the precise type of carbonate cannot be done unambiguously only by O-PTIR. On the basis of the SEM-EDX results (Fig. 1D), this could correspond to a type of potassium carbonate resulting from the degradation of the potash glass (*42*).

Spectrum 4 in Fig. 1B is characterized by an intense and broad absorption at around 1440 cm^−1^. The explanation for the broad and complex nature of the band can be found in the overlapping of the signals from several species, such as the ones in spectrum 2 and spectrum 3 in Fig. 1B, with additional metal carbonates and/or a mixed metal carbonates (*41*). The copresence of different species is in agreement with the overlapping of Zn, K, and Cu signals observed in these areas with SEM-EDX (Fig. 1D).

IR spectra with similar bands were observed in areas rich in Zn, K, and Cu also on a glass bead (Fig. 2A, spectrum 3) and on two corroded brass wires (Fig. 3A, spectrum 1, and fig. S10), although with slightly different features. In particular, a carbonate with a CO_3_^2−^ asymmetric stretching vibration at 1422 cm^−1^ and two bending vibrations at 876 and 845 cm^−1^ was observed in a white corrosion product on the surface of the glass bead (Fig. 2A, spectrum 3), while a carbonate with a CO_3_^2−^ asymmetric stretching vibration shifted to 1480 cm^−1^, and either one bending vibration at 878 cm^−1^ or two bending vibrations at 880 and 842 cm^−1^ were observed on two different brass wires (by O-PTIR, Fig. 3A, spectrum 1; and by SR-μFTIR, fig. S10, spectra 3 and 4).

In all cases, a more detailed interpretation of these complex mixtures of carbonates is further complicated by the overlap with the signals of Cu formate (particularly evident in fig. S10, spectra 2 to 4). Nonetheless, the existence of a spatial correlation between the carbonate signals identified by O-PTIR and the Zn, K, and, to a minor extent, Cu identified by SEM-EDX on the sequin, glass bead, and corroded wires (Figs. 1 to 3 and fig. S10, respectively) suggests that mixed carbonates might be present in all the samples. Although further studies would be necessary to fully characterize the nature of these carbonates, the identification of generic mixed carbonates most likely containing K, Zn, and possibly Cu on these samples is in itself remarkable. In order for these corrosion products to be formed, Zn^2+^, K^+^ (and Cu^2+^), and solubilized atmospheric CO_2_ would have to be simultaneously present in an alkaline solution [similarly to what described by Eggert *et al.* (*9*)]. Such a condition would have been likely met, in the Enclosed Gardens, only in the case of a direct contact and interaction between weathered potash glass (such as the one of the glass beads in analysis) and the brass alloy.

On the surface of the glass bead, an additional species was also observed in an area rich in Ca, K, Cu, and S (Fig. 2, spectrum 4). The high S content, together with the intense bands at 1120 cm^−1^ (SO_4_^2−^ stretching) and 1630 cm^−1^ (OH bending), suggests the presence of sulfates/hydrated sulfates [such as gypsum (*43*) or syngenite (*44*)], possibly mixed with a metal (probably Cu, Ca, K, or a mixture of these metals) carboxylate (additional peaks at ≈1600 cm^−1^ related to the C-O asymmetric stretching of COO^−^, and 1473 and 1448 cm^−1^ related to the C-O symmetric stretching of COO^−^ and to C-H vibrations). Sulfates and hydrated sulfates are often encountered as degradation products on the surface of potash-lime glass because of the interaction with atmospheric agents (*45*, *46*), while the presence of metal carboxylates in the degradation products might further support the hypothesis of a glass-induced corrosion of brass [Cu often forms complex mixed carboxylates under these conditions (*9*, *17*)].

### Additional considerations for the analysis of heritage objects by O-PTIR

Apart from the notable features described until now, a number of additional points need to be discussed when it comes to the application of O-PTIR to the study of cultural heritage. The first point regards the signal-to-noise ratio and the time of analysis. With five accumulations per point (total time of analysis around 1 min per point), the signal-to-noise ratio is high but remains lower than for the state-of-the-art SR-μFTIR, particularly in the 1200- to 800-cm^−1^ region. This did not represent a limitation in the context of this study, but, shall a higher signal quality be needed (e.g., when investigating materials with low IR absorption coefficients in complex mixtures), this could be easily improved by increasing the number of accumulations (keeping in mind that this would result in a longer measuring time).

Another important point regards the size of the sample chamber. Only objects that can fit the sample chamber of the instrument can, in fact, be analyzed noninvasively. In a standard configuration, this translates to a maximum size of 11(*x*) × 7.5(*y*) × 1.6(*z*) cm; however, the allowed range on the *z* axis can, in principle, be increased, opening to the analysis of larger artifacts. Even in the standard configuration used in this case, nonetheless, this technique is suitable for the study of different cultural heritage objects, such as not only fragments of illuminated manuscripts and papyri [where the importance of submicrometric measurements has been previously demonstrated (*47*, *48*)] but also jewels or other small multimaterial objects (which tend to form degradation compounds at the interfaces between materials and that could be problematic with other IR-based techniques because of their 3D shape and complexity).

Last, it is important to keep in mind that O-PTIR uses a visible laser in a similar fashion to μRaman, and this might represent a drawback for heritage materials (because any damage or irreversible transformation should be avoided). For this reason, the possibility of radiation damage for sensitive materials, which could potentially hamper further analysis by other techniques, should be always checked and the power of the probe laser optimized to minimize the risk. Likewise, also the power of the pump (IR) laser should be optimized to prevent the thermal damage of potentially sensitive materials. Nonetheless, as clearly shown for the sensitive samples considered in this study (fig. S8), by using optimized parameters, the actual risk remains much lower than for μRaman, thanks to the low laser power needed (both for pump and probe lasers) to obtain a clear response.

## DISCUSSION

While traditional characterization techniques such as XRPD and μRaman proved mostly unsuccessful for the study of the glass and metal samples in analysis, the application of O-PTIR (in combination with complementary techniques) allowed us to unravel information that would have otherwise remained hidden. O-PTIR made it possible to record IR spectra in a completely noninvasive and nondestructive manner, with high signal-to-noise ratios despite the lack of sample preparation. The low laser power needed to obtain results and the lack of interference from fluorescence allowed products that μRaman failed to identify to be characterized. Moreover, the high spatial resolution of the technique shed light on the extreme heterogeneity and on the spatial distribution of the corrosion products on the samples. More in detail, clear markers of glass-induced metal corrosion processes were identified thanks to O-PTIR on the surface of both metal and glass objects. In particular, basic copper formate [Cu_2_(OH)_3_(HCOO)] and different types of carbonates were found intimately mixed in the corrosion products.

This study represents the first attempt to include O-PTIR in a noninvasive multianalytical approach for the characterization of cultural heritage objects. It is clear from the results obtained that this novel technique can be easily combined with other commonly used methods for the analysis of heritage materials (μXRF, MA-XRF, SEM-EDX, and μCT) in a complementary approach, allowing to obtain detailed chemical information on the sample surface at a submicrometric level. Given the versatility, the lack of sample preparation, and high spatial resolution, together with the nondestructive and noncontact nature of the technique, O-PTIR has certainly a great potential to become a relevant tool in the future of cultural heritage analyses.

## MATERIALS AND METHODS

### Analytical methods

Exploratory MA-XRF, μXRF, and μCT analyses were performed to identify potential areas of interest on the single samples. The experimental details of these analyses are given in the Supplementary Materials.

O-PTIR measurements (spectra and images) were collected on the mIRage IR microscope (Photothermal Spectroscopy Corp.). Spectra were collected in reflection mode, 2-cm^−1^ spectral data point spacing, through a 40×, 0.78–numerical aperture, 8-mm working distance Schwarzschild objective (spot size of approximately 450 nm). The pump IR source was a pulsed, tunable four-stage QCL device, scanning from 800 to 1900 cm^−1^. The probe was a continuous wave (CW) 532-nm visible variable power laser. The power of both lasers was optimized before the analysis to not cause damage to the sample. Specifically, the QCL laser power was set to 100% (less than 3 mW on the sample), while the probe laser power was set to 0.25% (≈80 μW on the sample). To obtain a good signal-to-noise ratio to interpret the spectral difference, five spectra were averaged at each point, resulting in a total measuring time of approximately 1 min per point. The data treatment was performed using the Quasar 1.0.0 software (*49*, *50*). Spectral maps were generated by normalizing the spectra by the minimum-maximum method (using the most intense band) and plotting the integrated area of selected peaks (with a linear baseline computed using two wave numbers at the feet of the peak).

Raman spectroscopy measurements were performed by an XploRA Plus Microscope (Horiba) with a 100-mW 785-nm laser (the effective power used was always ≤1% to avoid beam damage), considering the effective range of 50 to 2000 cm^−1^. At each point, five 10-s accumulations were collected. In addition, a lower-wavelength laser (540 nm) was tested but showed intense fluorescence bands and was therefore not further used.

SR-μFTIR measurements were performed at the Spectroscopy and Microscopy in the Infrared using Synchrotron (SMIS) beamline at Synchrotron Soleil (France) using a Thermo Fisher NEXUS FTIR spectrometer Nicolet 5700 attached to a microscope Continuum XL. Small fragments of the corrosion products were extracted and squashed in a diamond compression cell. Measurements were done in transmission mode accumulating 256 scans at 4-cm^−1^ spectral resolution with a spot size of 10 × 10 μm^2^, and the wave number range is between 800 and 4000 cm^−1^. The O-PTIR, Raman, and FTIR spectra presented have not been corrected to avoid any kind of distortion.

The XRPD analysis was carried out with a custom-built diffraction setup in reflection geometry at a fixed incident angle of 10° between the x-ray source and sample. The x-ray source generates a beam of monochromatic Cu-Ka radiation (8.04 keV) with a photon flux of 2.9 × 10^8^ photons s^−1^ and a focal diameter of 0.15 mm. Multiple point measurements with an exposure time of 10 s pt.^−1^ were performed to account for the heterogeneous nature of the samples. A 2D single-photon counting PILATUS 200-K detector was used to register the emerging diffraction signals. The analysis of the data was performed with the in-house–developed software package XRDUA (*51*), while the structural information was obtained from the American Mineralogist Crystal Structure Database.

The microscopic observation of the samples was performed by optical microscopy and SEM. The OM observation was performed with a Nikon Eclipse LV100 microscope. The samples were also examined with a field emission gun–environmental scanning electron microscope (FEG-ESEM) equipped with an EDX detector (FEI Quanta 250, USA; at Antwerp electrochemical and analytical sciences lab (A-Sense Lab) and Electron microscopy for materials science (EMAT) research group, University of Antwerp) using an accelerating voltage of 20 kV, a takeoff angle of 30°, a working distance of 10 mm, and a sample chamber pressure of 10^−4^ Pa. Imaging was performed on the basis of secondary electrons and backscattered electrons. Different EDX maps were collected using a beam current of ≈0.5 nA, at different resolution, with pixel size values from 0.5 to 2.7 μm and dwell time from 0.1 to 10 ms per pixel. From these EDX maps, several EDX spectra were extracted for quantification. The dwell time of these spectra lies in the range of 1 to 7 s per spectrum. An overview of all the analytical techniques used on the single samples is given in table S1.

## Supplementary Materials

### This PDF file includes:

Supplementary Text Figs. S1 to S10 Tables S1 and S2 References
