# Supplementary material for: Novel optical photothermal infrared (O-PTIR) spectroscopy for the noninvasive characterization of heritage glass-metal objects
Source: Sci Adv. 2022 Mar 4;8(9):eabl6769. doi: 10.1126/sciadv.abl6769 (PMC8896789; doi:10.1126/sciadv.abl6769)
Supplement: Supplementary file 1 — Supplementary Text Figs. S1 to S10 Tables S1 and S2 References [file sciadv.abl6769_sm.pdf]

Science Advances
AAAS

Supplementary Materials for

Novel optical photothermal infrared (O-PTIR) spectroscopy for the noninvasive characterization of heritage glass-metal objects

Andrea Marchetti, Victoria Beltran, Gert Nuyts, Ferenc Borondics, Steven De Meyer, Marina Van Bos, Jakub Jaroszewicz, Elke Otten, Marjolijn Debulpaep, Karolien De Wael*

*Corresponding author. Email: karolien.dewael@uantwerpen.be

Published 4 March 2022, *Sci. Adv.* **8**, eabl6769 (2022)
DOI: 10.1126/sciadv.abl6769

This PDF file includes:

Supplementary Text
Figs. S1 to S10
Tables S1 and S2
References

Supplementary Text

Supplementary experimental details

The exploratory elemental analysis of the samples was performed by means of µXRF and MA-XRF spectroscopies. µXRF point analysis were performed with an Artax portable micro-XRF spectrometer (Bruker GmbH, Germany) equipped with a Rh-probe. The X-ray generator was operated at 50 kV and 600 µA, while the acquisition time was 300 s. The beam was focused on the analysis spot with the help of a laser and a camera, point analysis with a spotsize of approximately 70 µm were performed. The analysis of the XRF spectra, including the integration of the characteristic fluorescence signals, was carried out using PyMCA(*64*). The MA-XRF elemental imaging of the sequin in analysis was performed with an M6 Jetstream macro-XRF scanning spectrometer (Bruker GmbH, Germany) equipped with a Rh-probe. The X-ray generator was operated at 50 kV and 600 µA. The elemental imaging data were obtained with a 50 µm spotsize, a distance between measuring points of 40 µm and a dwell time of 40 ms. The data analysis, including the calculation of the elemental distribution maps, was performed with the software of the instrument.

The conservation state and distribution of the corrosion products on the glass samples was studied by means of micro computed tomography (µCT). The samples were initially scanned by means of a benchtop tomographic scanner SkyScan 1172 (Bruker, Belgium). Different X-ray tube voltage (40 kV or 100 kV) and current (250 µA and 100 µA) were applied depending on the attenuation properties of the single samples. 500 projection images were collected with a step size of 0.4 degrees and a final resolution between 4 and 8 µm, depending on the size of the sample. The latest SkyScan (Bruker, Belgium) softwares (NRecon, DataViewer, CTAn, and CTVox) were used to reconstruct and process images and to obtain virtual slices and volume rendering reconstructions. Higher resolution scans were obtained with a different X-ray tomographic system (MICRO XCT-400, Xradia - Zeiss), at 40 kV and 200 µA. 800 projection images were collected with a step size of 0.25 degrees and a final resolution between 2 and 4 µm, depending on the size of the sample. The volume was reconstructed with the instrument software and was then exported to Avizo Fire (FEI Visualization Sciences Group) for further 3D image analysis.

Supplementary experimental results (exploratory elemental and CT analyses)

The elemental composition of the corroded areas of the metal objects was first studied by means of MA-XRF and µ-XRF. The results of the MA-XRF analysis (Fig. S2A and S2B), performed only on the sequin (sample 1S) it being the only relatively flat and large sample, confirmed the presence of Cu and Zn (main components of the brass alloy) in the bulk of the material. The analysis also clearly highlighted a close link between the distribution of elements such as K, Zn and to a minor extent Ca, and the severe corrosion observed (Fig. S2A and S2B). The abundant presence of K only in the corroded areas of the sequin was confirmed also by the SEM-EDX analysis, which also excluded the presence of significant amounts of other light elements (too light to be detected by XRF), such as Na (Fig. S2C and S2D). In a similar fashion, K was also systematically found in the corroded areas of the brass wires in analysis (Fig. S3A), often in association with higher Zn concentrations (Fig. S3B). The presence of relatively higher amounts of Zn in the degraded areas is expected for brass, since this less-noble component of the alloy is often preferentially leached out from the bulk metal (a phenomenon known as dezincification, although Cu leaching can also simultaneously take place) and, if not washed away, reprecipitated in oxidation products on the surface of the material(*65*, *66*). The systematic presence of K, on the other hand, is likely a result of the interaction with weathered historical glass, as described in the main body of the manuscript (*9*, *15*, *17*).

The non-invasive µXRF analysis of the glass samples showed a very similar composition for the two beads considered. In particular, both are made of a potash glass with a limited lime content, as indicated by the high K/Ca ratio (average=1.6±0.3) and by the low contribution of Pb in the

XRF spectrum (Fig. S4A, B)(*67*, *68*). It is important to mention that the experimental K/Ca ratio is likely affected by the poor conservation state of the glass, since the degradation is normally associated with the leaching of alkali ions from the surface. However, this process would cause a decrease of the ratio due to the leaching of K rather than an increase, hence not changing the attribution of the samples in analysis to the potash glass category. Particularly interesting is the presence of small amounts of Co (Fig. S4D), likely responsible for the dark blue color of the beads. Moreover, significant amounts of both Cu and Zn are observed in localized areas on the surface of the beads (Fig. S4E).

The SEM-EDX analysis performed on the surface of the glass beads confirmed both the overall composition of the objects (presence of K and Ca but absence of Na in significant amounts), as well as the high content of Cu and Zn, in particular in areas where green-blue corrosion products are clearly present (Fig. 2B). This suggests that these last two metals, main components of the brass objects, might be leached out from the metal and re-precipitated on the surface of the glass beads due to glass-induced metal corrosion processes(*9*, *17*).

To further test this hypothesis, the distribution of the Cu- and Zn-containing products on the surface of the glass beads was non-invasively investigated by means of µCT (Fig. S6). Since these corrosion products contain higher concentrations of (relatively) heavy atoms, characterized by higher attenuating properties compared to the lighter atoms in the silica network of the glass, they are in fact well-distinguishable in the final reconstructed volume. The results clearly showed that heavier corrosion products are distributed mainly on the surface of the central shaft (Fig. S6A) and on the inner rim of the beads (Fig. S6B). These are areas where the glass would have been mostly protected from surface deposition and where it certainly came in contact with a metallic wire (ubiquitously employed to assemble the decorative elements in the enclosed gardens(*8*)). Therefore, these are areas in which an interaction between metal and glass would have been more likely to take place.

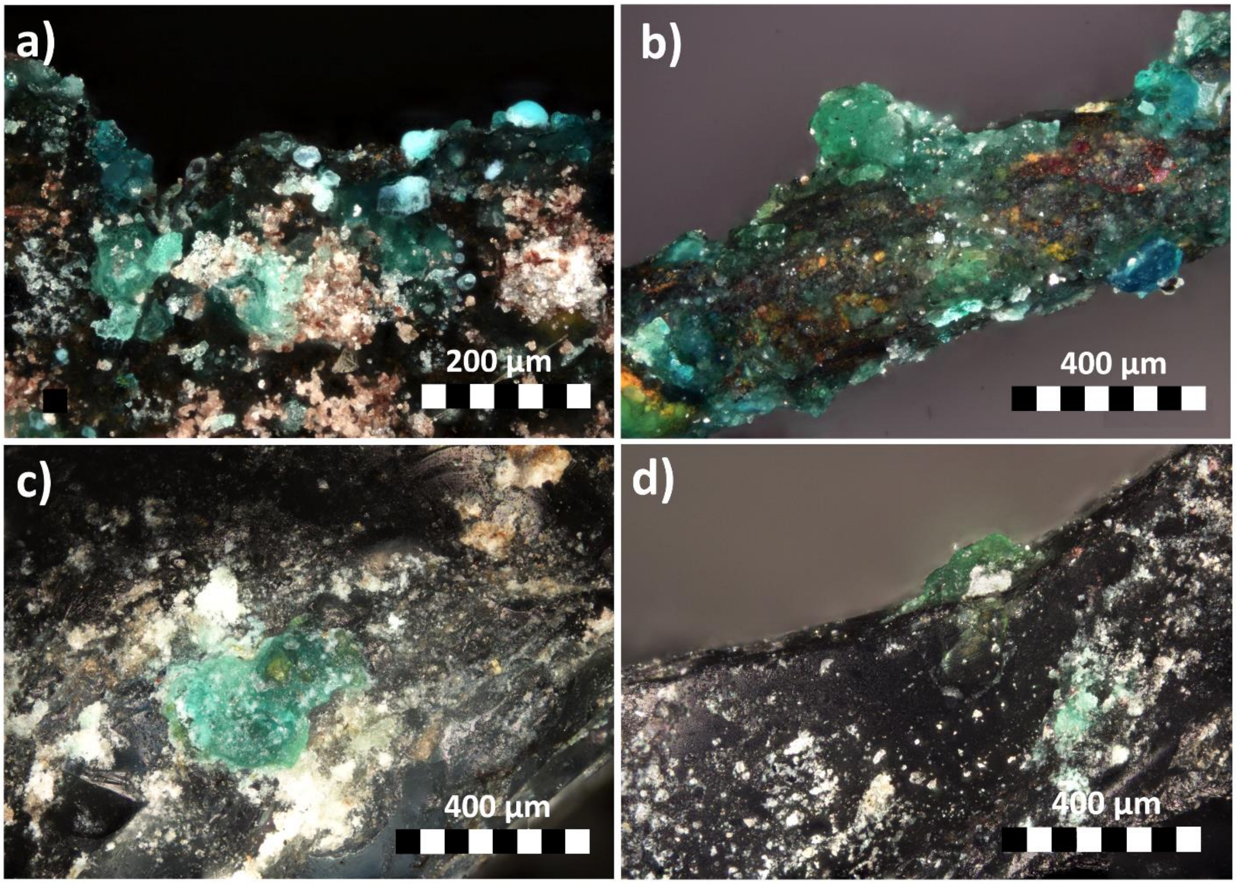

Fig. S1. Photomicrographs of the corrosion products on the surface of the samples (OM, bright field with polarized light). Examples of a) corroded brass sequin (sample 1S), b) corroded brass wire (sample 3W), c) glass bead (sample 1B), d) glass bead (sample 2B).

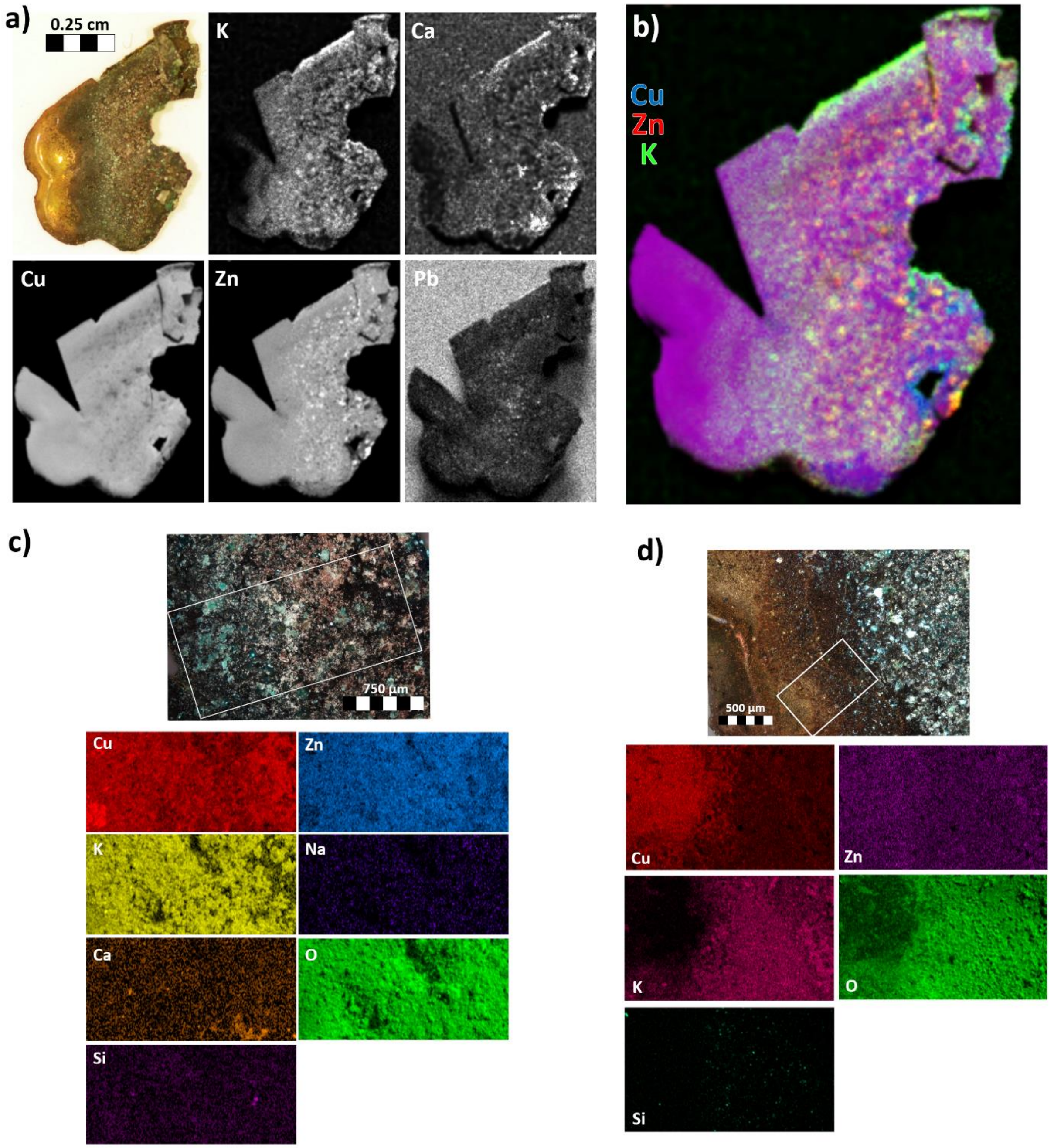

Fig. S2. Elemental analysis of the corroded sequin (sample 1S). a) MA-XRF single elemental maps; b) MA-XRF composite elemental map for Cu, Zn and K; c), d) SEM-EDX elemental maps. After the picture of the sample was taken (part a, top left) and before the elemental MA-XRF analysis were performed, a small triangular fragment of this object (the missing section visible in the elemental maps) was removed to be studied in the context of a different project. This is the reason for the differences in shape observed for the sequin in part a and b of the figure.

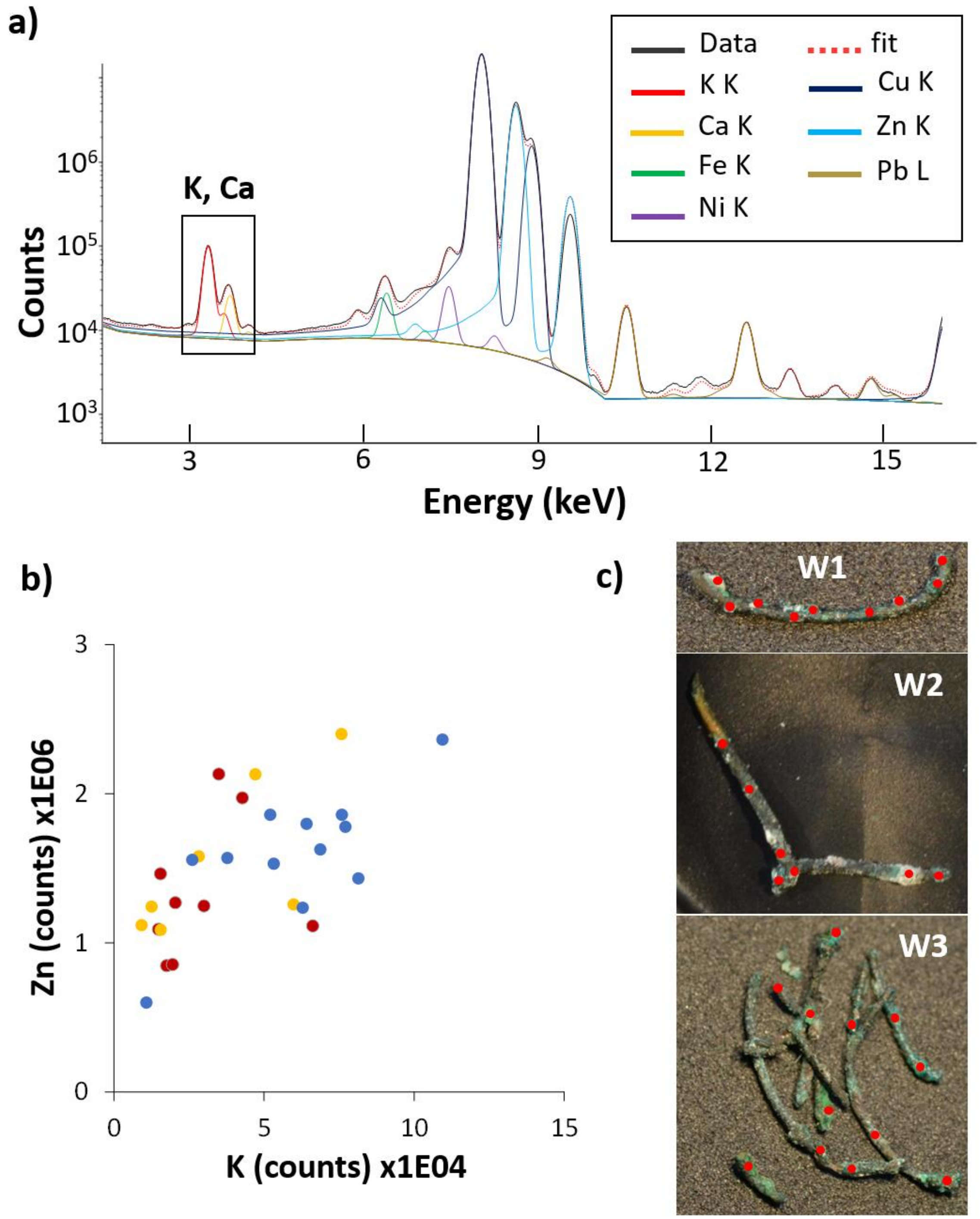

Fig. S3. µXRF analysis of the visually degraded areas of all the corroded brass wires analyzed. a) sum spectrum, in evidence the contribution of the most relevant elements present (in addition to the element shown, minor contributions of S, Cl, Ar, Mn, As, Rb, Sr, Rh, Sn and Hg are also found in the spectrum); b) elemental scatter plot for Zn and K (integrated K Lines; red = sample 1W, yellow= sample 2W, blue = sample 3W); c) approximate location of the areas analyzed.

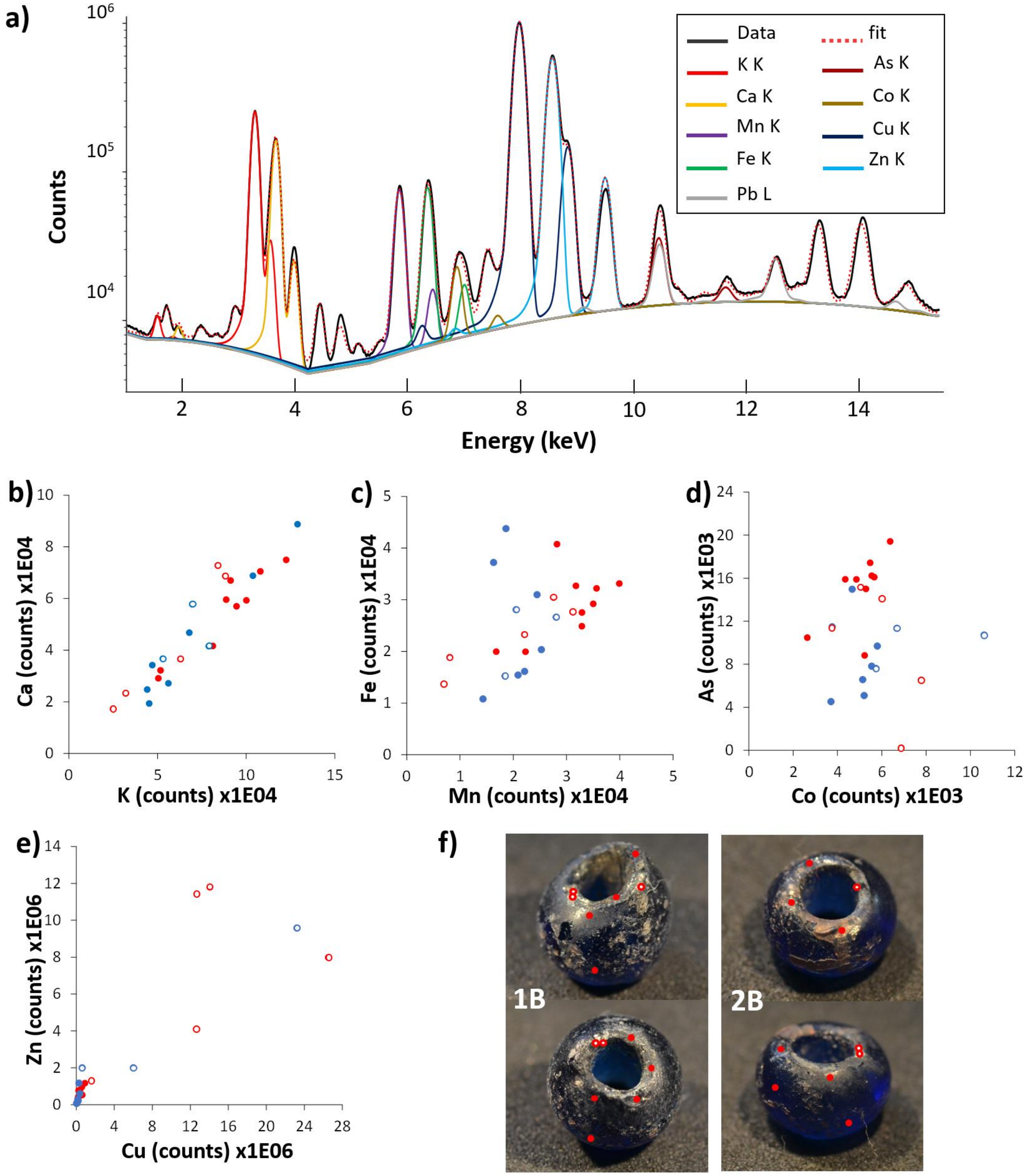

Fig. S4. µXRF analysis of different areas of the glass beads. a) sum spectrum, in evidence the contribution of relevant elements to the spectrum (in addition to the element shown, minor contributions of S, Cl, Ar, Ni, Rb, Sr, Rh, Sn and Hg are also found in the spectrum); b), c), d), e) elemental scatter plots (integrated K lines; red = sample 1B; blue = sample 2B; empty circles = areas with visible green-blue corrosion products); f) approximate location of the areas analyzed (red-white circles = areas with visible green-blue corrosion products).

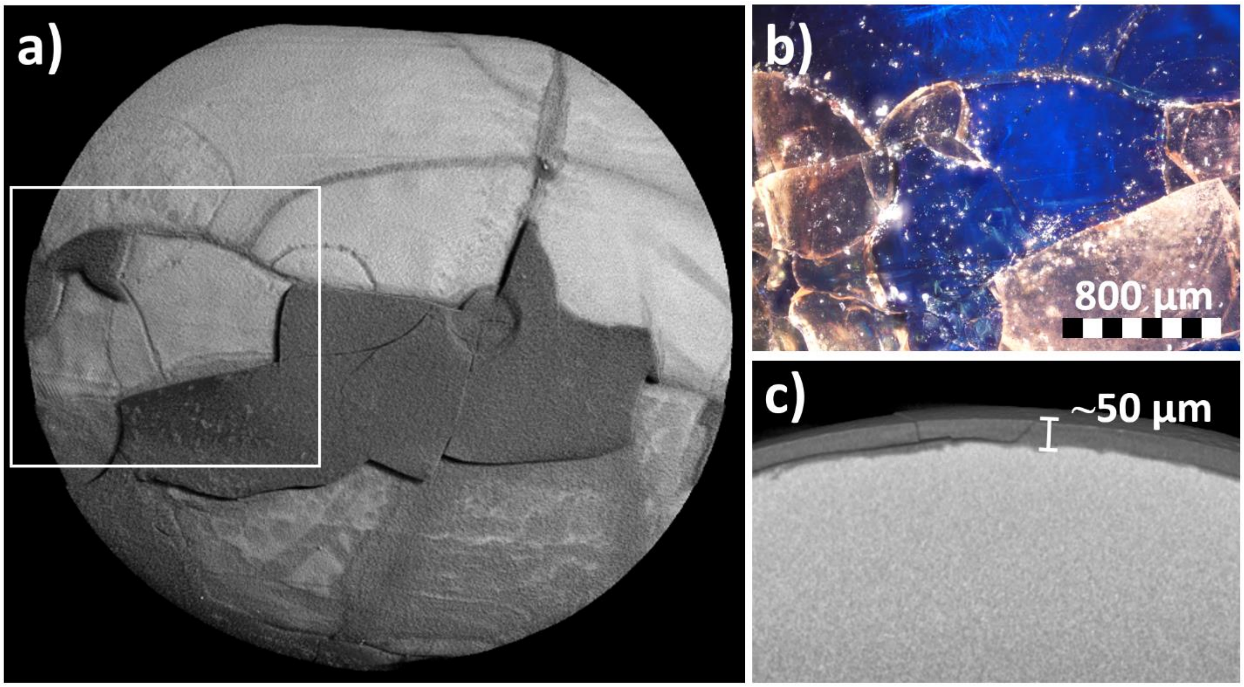

Fig. S5. Evidence of a partially detached iridescent gel layer on the surface of a glass bead (2B). a) µCT reconstructed volume, the less electronically dense gel layer appears darker than the healthy glass; b) photomicrograph (OM, bright field with polarized light); c) virtual cross-section of the gel layer clearly showing the partial detachment and the thickness of the layer.

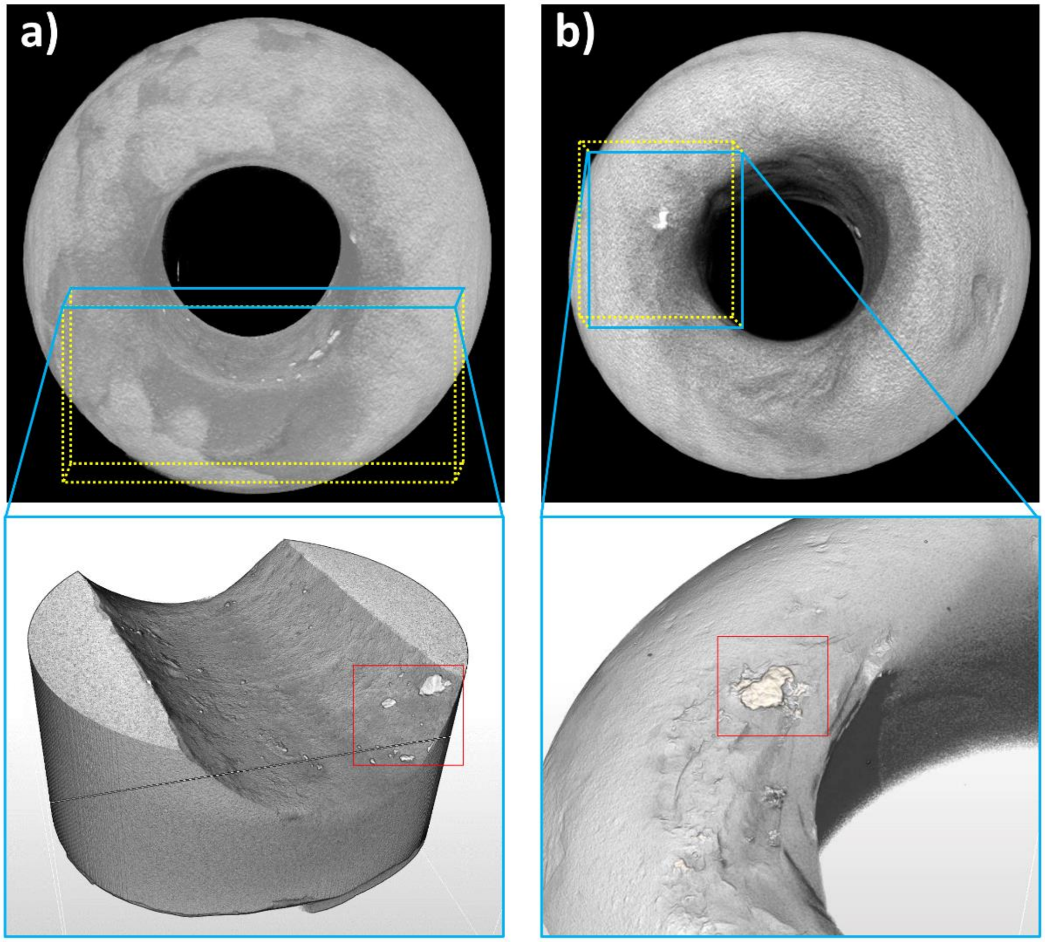

Fig. S6. μCT scan results. Reconstructed volume and localization of highly attenuating corrosion products (red rectangles) on the inner rim and central shaft of the two glass beads a) sample 2B, b) sample 1B, the region highlighted in red corresponds to the area analyzed in Fig. 2 in the main body of the manuscript.

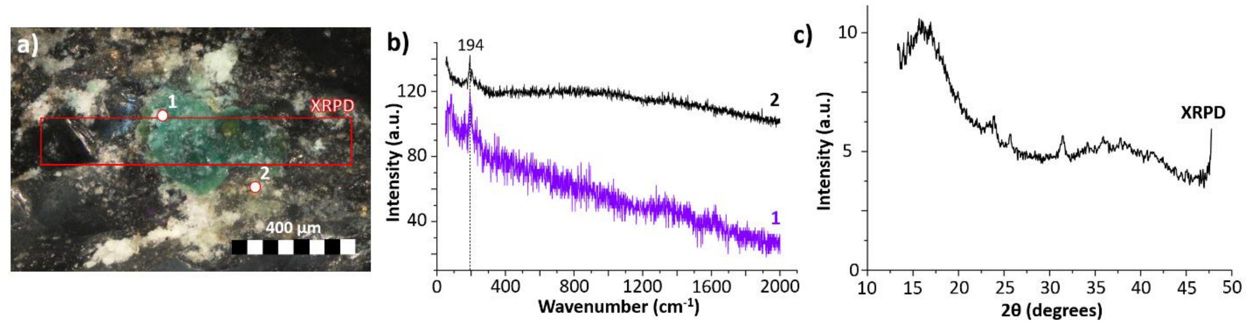

Fig. S7. Examples of the μRaman and MA-XRPD results obtained for the glass beads and wires (in this case the glass bead P1). a) OM photomicrographs and approximate location of the measurements (the region of interest is the same as in Figure 2 in the main body of the manuscript; b) μRaman spectra collected in point 1 and 2 (power: <1%); c) MA-XRPD result for the area in analysis. The low signal-to-noise ratio did not allow to perform any clear interpretation of the experimental results.

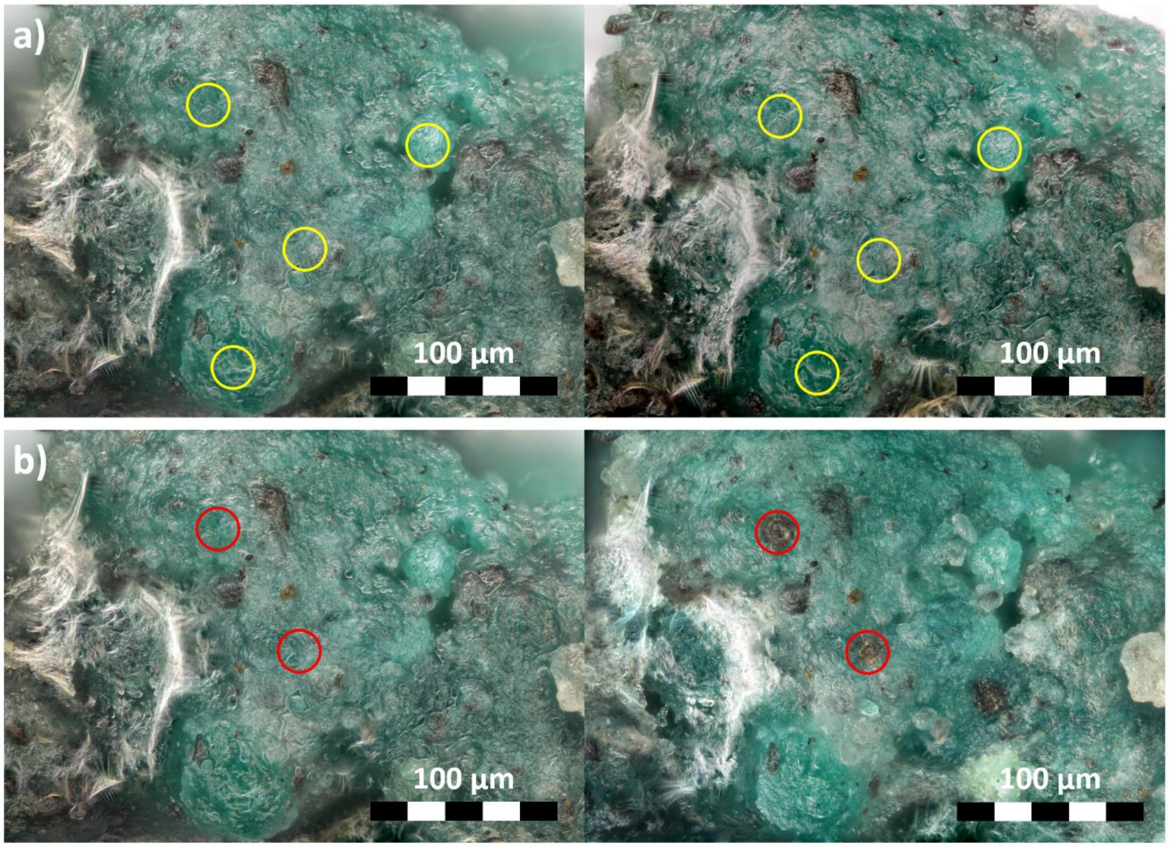

Fig. S8. OM photomicrograph of a corroded area of a brass wire (3W) before and after the analysis. a) O-PTIR (yellow circles, 10 replicates per point) and b) µRaman (red circles, single analysis, power: 1%)).

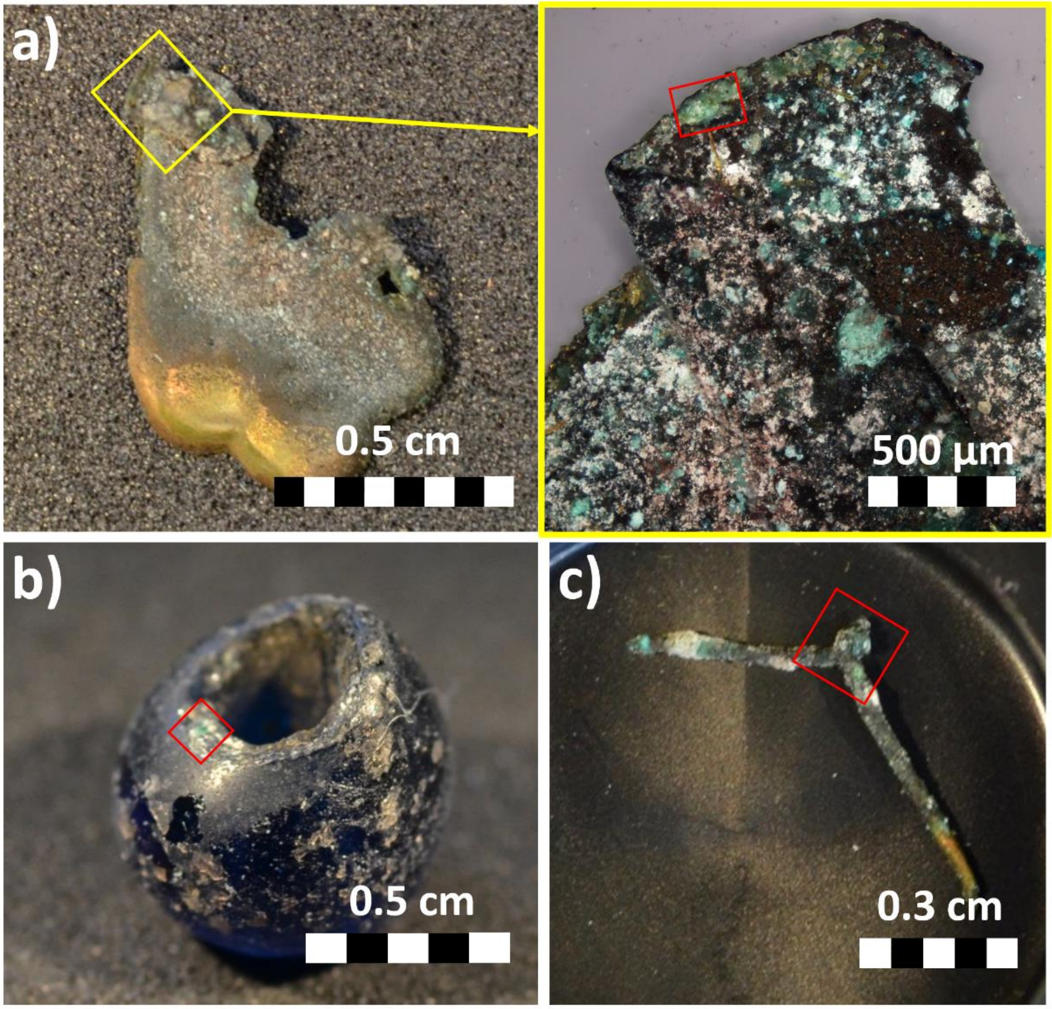

Fig. S9. Regions of interest (red rectangles) for the O-PTIR analysis presented in Figure 1, 2 and 3 respectively in the main body of the manuscript. a) sample 1S, b) sample 1B, c) sample 2W.

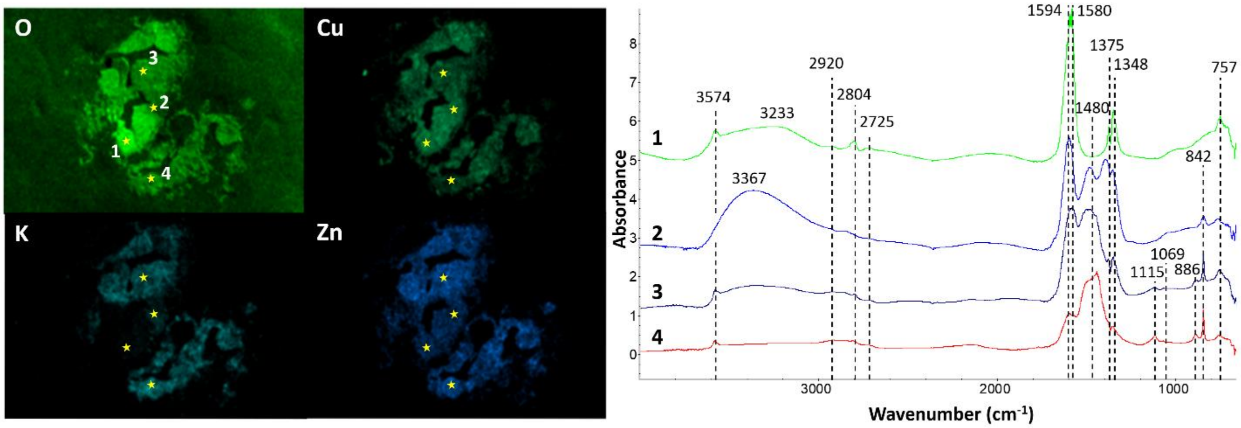

Fig. S10. SEM-EDX elemental maps and SR-FTIR spectra of the degradation products scratched from the surface of one corroded wire (3W). The material was pressed in a diamond compression cell prior to the analysis. In evidence the spots analyzed with SR-FTIR and the most intense absorption peaks in the spectra.

Table S1. Overview of the analytical techniques employed on the single samples.

	Code	µXRF	MA-XRF	SEM-EDX	µCT	µFTIR	MA-XRPD	µRaman	O-PTIR
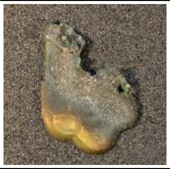	1S	X	X	X			X	X	X
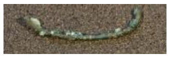	1W	X					X	X	X
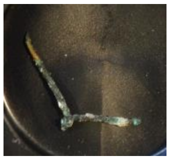	2W	X		X			X	X	X
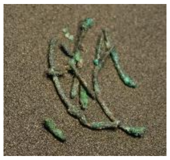	3W	X		X		X	X	X	X
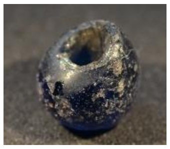	1B	X		X	X		X	X	X
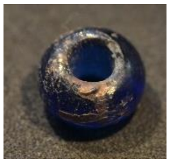	2B	X		X	X			X	X

Table S2. Overview of the spectral markers of the main compounds identified(*28*, *36*, *41*, *69*, *70*).

Compound	IR spectral markers (cm^{-1})	
Glass	1250 - 980	Si-O-Si and Si-O stretching
Carboxylate	1610 - 1550	C=O (asymmetric C-O stretching)
	1420 - 1300	C=O (symmetric C-O stretching)
Cu Formate	2800	CH (C-H stretching)
	1600	C=O (asymmetric C-O stretching)
	1377	CH (in-plane C-H bending)
	1350	C=O (symmetric C-O stretching)
Carbonate	1490 - 1410	CO_3^{2-} (C-O stretching)
	880 - 840	CO_3^{2-} (C-O bending)
Oxalate	1722 - 1615	$C_2O_4^{2-}$
	1410-1300	$C_2O_4^{2-}$
Sulfate	1120	SO_4^{2-} stretching

REFERENCES AND NOTES

1. M. Cotte, A. Genty-Vincent, K. Janssens, J. Susini, Applications of synchrotron x-ray nano-probes in the field of cultural heritage. *C. R. Phys.* **19**, 575–588 (2018).

2. J. M. Madariaga, Analytical chemistry in the field of cultural heritage. *Anal. Methods* **7**, 4848–4876 (2015).

3. J. A. Reffner, Advances in infrared microspectroscopy and mapping molecular chemical compositionat submicrometer spatial resolution. *Spectroscopy* **33**, 12–17 (2018).

4. N. Baden, H. Kobayashi, N. Urayama, Submicron-resolution polymer orientation mapping by optical photothermal infrared spectroscopy. *Int. J. Polym. Anal. Charact.* **25**, 1–7 (2020).

5. O. Klementieva, C. Sandt, I. Martinsson, M. Kansiz, G. K. Gouras, F. Borondics, Super-resolution infrared imaging of polymorphic amyloid aggregates directly in neurons. *Adv. Sci.* **7**, 1903004 (2020).

6. V. Beltran, A. Marchetti, G. Nuyts, M. Leeuwestein, C. Sandt, F. Borondics, K. De Wael, Nanoscale analysis of historical paintings by means of O-PTIR spectroscopy: The identification of the organic particles in L′Arlésienne (portrait of Madame Ginoux) by Van Gogh. *Angew. Chem. Int. Ed. Engl.* **60**, 22753– 22760 (2021).

7. B. Hofjes, *Museum Hof van Busleyden* www.hofvanbusleyden.be/besloten-hofjes.

8. L. Watteeuw, H. Iterbeke, *Enclosed Gardens of Mechelen. Late Medieval Paradise Gardens Revealed* (Amsterdam Univ. Press, Amsterdam, 2018).

9. G. Eggert, Corroding glass, corroding metals: Survey of joint metal/glass corrosion products on historic objects. *Corros. Eng. Sci. Technol.* **45**, 414–419 (2010).

10. H. Scholze, Chemical durability of glasses. *J. Non Cryst. Solids* **52**, 91–103 (1982).

11. M. Schreiner, Glass of the past: The degradation and deterioration of medieval glass artifacts. *Mikrochim. Acta* **104**, 255–264 (1991).

12. B. C. Bunker, Molecular mechanisms for corrosion of silica and silicate glasses. *J. Non Cryst. Solids* **179**, 300–308 (1994).

13. O. Majérus, P. Lehuédé, I. Biron, F. Alloteau, S. Narayanasamy, D. Caurant, Glass alteration in atmospheric conditions: Crossing perspectives from cultural heritage, glass industry, and nuclear waste management. *NPJ Mater. Degrad.* **4**, 27 (2020).

14. S. Cagno, G. Nuyts, S. Bugani, K. De Vis, O. Schalm, J. Caen, L. Helfen, M. Cotte, P. Reischig, K. Janssens, Evaluation of manganese-bodies removal in historical stained glass windows via SR-μ-XANES/XRF and SR-μ-CT. *J. Anal. At. Spectrom* **26**, 2442–2451 (2011).

15. D. Rohanová, H. Hradecká, L. Klikarová, P. Hauková, S. Švarcová, Leach testing of model glasses containing sodium or potassium ions as a flux. *Glas. Technol. Eur. J. Glas. Sci. Technol. Part A.* **53**, 109–117 (2012).

16. A. Fischer, G. Eggert, R. Dinnebier, T. Runčevski, When glass and metal corrode together, V: Sodium copper formate. *Stud. Conserv.* **63**, 342–355 (2018).

17. A. Fischer, G. Eggert, J. Stelzner, S. Bette, R. E. Dinnebier, in *METAL 2019. Proceedings of the Interim Meeting of the ICOM-CC Metals Working Group*, C. Chemello, L. Brambilla, E. Joseph, Eds. (ICOM-CC, HE-Arc CR, Neuchatel, Switzerland, 2019), pp. 158–167.

18. S. Bette, G. Eggert, A. Fischer, R. E. Dinnebier, Glass-induced lead corrosion of heritage objects: Structural characterization of K(OH)·2PbCO3. *Inorg. Chem.* **56**, 5762–5770 (2017).

19. A. Fischer, G. Eggert, D. Kirchner, When Glass and Metal Corrode Together, IV: Sodium Lead Carbonate Hydroxide. *Interim Meet. ICOM-CC Met. Gr. 2013 Edinburgh, Scotl.*, 13–19 (2013).

20. W. Anaf, L. Bencs, R. Van Grieken, K. Janssens, K. De Wael, Indoor particulate matter in four Belgian heritage sites: Case studies on the deposition of dark-colored and hygroscopic particles. *Sci. Total Environ.* **506–507**, 361–368 (2015).

21. M. P. Ligocki, L. G. Salmon, T. Fall, M. C. Jones, W. W. Nazaroff, G. R. Cass, Characteristics of airborne particles inside southern California museums. *Atmos. Environ. A Gen. Top.* **27**, 697–711 (1993).

22. J. Grau-Bové, M. Strlič, Fine particulate matter in indoor cultural heritage: A literature review. *Herit. Sci.* **1**, 8–17 (2013).

23. C. R. Kurkjian, W. R. Prindle, Perspectives on the history of glass composition. *J. Am. Ceram. Soc.* **81**, 795–813 (1998).

24. B. Gratuze, I. Pactat, N. Schibille, Changes in the signature of cobalt colorants in late antique and early Islamic glass production. *Minerals* **8**, 225 (2018).

25. H. Euler, B. Barbier, A. Kirfel, S. Haseloff, G. Eggert, Crystal structure of trihydroxydicopper formate, $Cu_2(OH)_3(HCOO)$. *Z. Kristallogr.* **224**, 609–610 (2009).

26. A. Fischer, G. Eggert, J. Stelzner, When glass and metal corrode together, VI: Chalconatronite. *Stud. Conserv.* **65**, 152–159 (2020).

27. F. Vanmeert, W. De Nolf, S. De Meyer, J. Dik, K. Janssens, Macroscopic x-ray powder diffraction scanning, a new method for highly selective chemical imaging of works of art: Instrument optimization. *Anal. Chem.* **90**, 6436–6444 (2018).

28. A. Rodrigues, S. Fearn, M. Vilarigues, Historic K-rich silicate glass surface alteration: Behaviour of high-silica content matrices. *Corros. Sci.* **145**, 249–261 (2018).

29. K. Ito, H. J. Bernstein, The vibrational spectra of the formate, acetate, and oxalate ions. *Can. J. Chem.* **34**, 170–178 (1956).

30. K. Nakamoto, Infrared and Raman spectra of inorganic and coordination compounds. *Handb. Vib. Spectrosc.*, 1872–1892 (2006).

31. R. L. Frost, W. N. Martens, D. L. Wain, M. C. Hales, Infrared and infrared emission spectroscopy of the zinc carbonate mineral smithsonite. *Spectrochim. Acta A Mol. Biomol. Spectrosc.* **70**, 1120–1126 (2008).

32. C. E. Weir, E. R. Lippincott, Infrared studies of aragonite, calcite, and vaterite type structures in the borates, carbonates, and nitrates. *J. Res. Natl. Bur. Stand. A Phys. Chem.* **65A**, 173–180 (1961).

33. L. Robinet, K. Eremin, B. Cobo Del Arco, L. T. Gibson, A Raman spectroscopic study of pollution-induced glass deterioration. *J. Raman Spectrosc.* **35**, 662–670 (2004).

34. I. F. Kadikova, E. A. Morozova, T. V. Yuryeva, I. A. Grigorieva, V. A. Yuryev, Study of deteriorating semiopaque turquoise lead-potassium glass beads at different stages of corrosion using micro-FTIR spectroscopy. arXiv: 1705.09394 (2017).

35. L. Rampazzi, Calcium oxalate films on works of art: A review. *J. Cult. Herit.* **40**, 195–214 (2019).

36. S. Hosseinpour, M. Forslund, C. M. Johnson, J. Pan, C. Leygraf, Atmospheric corrosion of Cu, Zn, and Cu-Zn alloys protected by self-assembled monolayers of alkanethiols. *Surf. Sci.* **648**, 170–176 (2016).

37. R. O. Carter, B. D. Poindexter, W. H. Weber, Vibrational spectra of copper formate tetrahydrate, copper formate dihydrate and three anhydrous forms of copper formate. *Anal. Chim. Acta* **2**, 125–134 (1991).

38. R. S. Krishnan, P. S. Ramanujam, Raman and Infrared Spectra of copper formate tetrahydrate. *Spectrochim. Acta A* **28A**, 2227–2231 (1972).

39. E. A. Secco, Spectroscopic properties of SO4 (and OH) in different molecular and crystalline environments. I. Infrared spectra of Cu4(OH)6SO4, Cu4(OH)4OSO4, and CU3(OH)4SO4. *Can. J. Chem.* **66**, 329–336 (1988).

40. L. T. Gibson, C. M. Watt, Acetic and formic acids emitted from wood samples and their effect on selected materials in museum environments. *Corros. Sci.* **52**, 172–178 (2010).

41. G. C. Jones, B. Jackson, *Infrared Transmission Spectra of Carbonate Minerals* (Springer, Dordrecht, 1993).

42. M. Vilarigues, R. C. da Silva, The effect of Mn, Fe and Cu ions on potash-glass corrosion. *J. Non. Cryst. Solids* **355**, 1630–1637 (2009).

43. J. Kiefer, A. Stärk, A. L. Kiefer, H. Glade, Infrared spectroscopic analysis of the inorganic deposits from water in domestic and technical heat exchangers. *Energies* **11**, 798 (2018.

44. J. T. Kloprogge, R. D. Schuiling, Z. Ding, L. Hickey, D. Wharton, R. L. Frost, Vibrational spectroscopic study of Syngenite formed during the treatment of liquid manure with sulphuric acid. *Vib. Specroscopy.* **28**, 209–221 (2002).

45. L. Gentaz, T. Lombardo, A. Chabas, C. Loisel, A. Verney-Carron, Impact of neocrystallisations on the SiO2–K2O–CaO glass degradation due to atmospheric dry depositions. *Atmos. Environ.* **55**, 459–466 (2012).

46. M. Melcher, M. Schreiner, Statistical evaluation of potash-lime-silica glass weathering. *Anal. Bioanal. Chem.* **379**, 628–639 (2004).

47. T. Christiansen, M. Cotte, W. de Nolf, E. Mouro, J. Reyes-Herrera, S. de Meyer, F. Vanmeert, N. Salvadó, V. Gonzalez, P. E. Lindelof, K. Mortensen, K. Ryholt, K. Janssens, S. Larsen, Insights into the composition of ancient Egyptian red and black inks on papyri achieved by synchrotron-based microanalyses. *Proc. Natl. Acad. Sci. U.S.A.* **117**, 27825–27835 (2020).

48. D. Kurouski, S. Zaleski, F. Casadio, R. P. Van Duyne, N. C. Shah, Tip-enhanced raman spectroscopy (TERS) for in situ identification of indigo and iron gall ink on paper. *J. Am. Chem. Soc.* **136**, 8677–8684 (2014).

49. M. Toplak, G. Birarda, S. Read, C. Sandt, S. M. Rosendahl, L. Vaccari, J. Demšar, F. Borondics, Infrared orange: Connecting hyperspectral data with machine learning. *Synchrotron Radiat. News* **30**, 40–45 (2017).

50. J. Demšar, T. Curk, A. Erjavec, Č. Gorup, T. Hočevar, M. Milutinovič, M. Možina, M. Polajnar, M. Toplak, A. Starič, M. Štajdohar, L. Umek, L. Žagar, J. Žbontar, M. Žitnik, B. Zupan, Orange: Data mining toolbox in python. *J. Mach. Learn. Res.* **14**, 2349–2353 (2013).

51. W. De Nolf, F. Vanmeert, K. Janssens, XRDUA: Crystalline phase distribution maps by two-dimensional scanning and tomographic (micro) x-ray powder diffraction. *J. Appl. Cryst.* **47**, 1107–1117 (2014).

52. O. Antonoglou, J. Moustaka, I. D. S. Adamakis, I. Sperdouli, A. A. Pantazaki, M. Moustakas, C. Dendrinou-Samara, Nanobrass CuZn nanoparticles as foliar spray nonphytotoxic fungicides. *ACS Appl. Mater. Interfaces* **10**, 4450–4461 (2018).

53. V. Gonzalez, M. Cotte, F. Vanmeert, W. de Nolf, K. Janssens, X-ray diffraction mapping for cultural heritage science: A review of experimental configurations and applications. *Chem. Eur. J.* **26**, 1703–1719 (2020).

54. L. Bertrand, L. Robinet, M. Thoury, K. Janssens, S. X. Cohen, S. Schöder, Cultural heritage and archaeology materials studied by synchrotron spectroscopy and imaging. *Appl. Phys. A* **106**, 377–396 (2012).

55. L. Monico, M. Cotte, F. Vanmeert, L. Amidani, K. Janssens, G. Nuyts, J. Garrevoet, G. Falkenberg, P. Glatzel, A. Romani, C. Miliani, Damages induced by synchrotron radiation-based x-ray microanalysis in chrome yellow paints and related Cr-compounds: Assessment, quantification, and mitigation strategies. *Anal. Chem.* **92**, 14164–14173 (2020).

56. M. Cotte, P. Dumas, Y. Taniguchi, E. Checroun, P. Walter, J. Susini, Recent applications and current trends in Cultural Heritage Science using synchrotron-based Fourier transform infrared micro-spectroscopy. *C. R. Phys.* **10**, 590–600 (2009).

57. N. Salvadó, S. Butí, M. J. Tobin, E. Pantos, A. J. N. W. Prag, T. Pradell, Advantages of the use of SR-FT-IR microspectroscopy: Applications to cultural heritage. *Anal. Chem.* **77**, 3444–3451 (2005).

58. N. Salvadó, S. Butí, J. Nicholson, H. Emerich, A. Labrador, T. Pradell, Identification of reaction compounds in micrometric layers from gothic paintings using combined SR-XRD and SR-FTIR. *Talanta* **79**, 419–28 (2009).

59. G. D. Smith, L. Burgio, S. Firth, R. J. H. Clark, Laser-induced degradation of lead pigments with reference to Botticelli's Trionfo d'Amore. *Anal. Chim. Acta* **440**, 185–188 (2001).

60. A. De Santis, E. Mattei, C. Pelosi, Micro-Raman and stratigraphic studies of the paintings on the 'Cembalo' model musical instrument (A.D. 1650) and laser-induced degradation of the detected pigments. *J. Raman Spectrosc.* **38**, 1368–1378 (2007).

61. M. C. Caggiani, P. Colomban, Raman microspectroscopy for Cultural Heritage studies. *Phys. Sci. Rev.* **3**, 1–18 (2019).

62. X. Ma, V. Beltran, G. Ramer, G. Pavlidis, D. Y. Parkinson, M. Thoury, T. Meldrum, A. Centrone, B. H. Berrie, Revealing the distribution of metal carboxylates in oil paint from the micro- to nanoscale. *Angew. Chem. Int. Ed.* **58**, 11652–11656 (2019).

63. S. Morsch, B. A. Van Driel, K. J. Van Den Berg, J. Dik, Investigating the photocatalytic degradation of oil paint using ATR-IR and AFM-IR. *ACS Appl. Mater. Interfaces* **9**, 10169–10179 (2017).

64. V. A. Solé, E. Papillon, M. Cotte, P. Walter, J. Susini, A multiplatform code for the analysis of energy-dispersive x-ray fluorescence spectra. *Spectrochim. Acta Part B At. Spectrosc.* **62**, 63–68 (2007).

65. S. Goidanich, J. Brunk, G. Herting, M. A. Arenas, I. Odnevall Wallinder, Atmospheric corrosion of brass in outdoor applications. Patina evolution, metal release and aesthetic appearance at urban exposure conditions. *Sci. Total Environ.* **412–413**, **46**–57 (2011).

66. P. Zhou, M. J. Hutchison, J. W. Erning, J. R. Scully, K. Ogle, An in situ kinetic study of brass dezincification and corrosion. *Electrochim. Acta* **229**, 141–154 (2017).

67. O. Schalm, K. Janssens, H. Wouters, D. Caluwé, Composition of 12-18th century window glass in Belgium: Non-figurative windows in secular buildings and stained-glass windows in religious buildings. *Spectrochim. Acta Part B At. Spectrosc.* **62**, 663–668 (2007).

68. S. Cagno, G. Van der Snickt, S. Legrand, J. Caen, M. Patin, W. Meulebroeck, Y. Dirkx, M. Hillen, G. Steenackers, A. Rousaki, P. Vandenabeele, K. Janssens, Comparison of four mobile, non-invasive diagnostic techniques for differentiating glass types in historical leaded windows: MA-XRF, UV–Vis–NIR, Raman spectroscopy and IRT. *Xray Spectrom.* **50**, 293–309 (2021).

69. R. L. Frost, J. Yang, Z. Ding, Raman and FTIR spectroscopy of natural oxalates: Implications for the evidence of life on Mars. *Chin. Sci. Bull.* **48**, 1844–1852 (2003).

70. L. J. Bellamy, *The Infra-Red Spectra of Complex Molecules* (Springer Science & Business Media, 2013).
